# Domain generalization for image classification based on simplified self ensemble learning

**DOI:** 10.1371/journal.pone.0320300

**Published:** 2025-04-04

**Authors:** Zhenkai Qin, Xinlu Guo, Jun Li, Yue Chen

**Affiliations:** 1 College of Information Technology, Guangxi Police College, Nanning,China; 2 College of Information Engineering, China Jiliang University, Hangzhou,China; 3 College of Continuing Education, Guangxi Police College, Nanning,China; 4 College of Information Technology, Guangxi Police College, Nanning,China; South China University of Technology, CHINA

## Abstract

Domain generalization seeks to acquire knowledge from limited source data and apply it to an unknown target domain. Current approaches primarily tackle this challenge by attempting to eliminate the differences between domains. However, as cross-domain data evolves, the discrepancies between domains grow increasingly intricate and difficult to manage, rendering effective knowledge transfer across multiple domains a persistent challenge. While existing methods concentrate on minimizing domain discrepancies, they frequently encounter difficulties in maintaining effectiveness when confronted with high data complexity. In this paper, we present an approach that transcends merely eliminating domain discrepancies by enhancing the model’s adaptability to improve its performance in unseen domains. Specifically, we frame the problem as an optimization process with the objective of minimizing a weighted loss function that balances cross-domain discrepancies and sample complexity. Our proposed self-ensemble learning framework, which utilizes a single feature extractor, simplifies this process by alternately training multiple classifiers with shared feature extractors. The introduction of focal loss and complex sample loss weight further fine-tunes the model’s sensitivity to hard-to-learn instances, enhancing generalization to difficult samples. Finally, a dynamic loss adaptive weighted voting strategy ensures more accurate predictions across diverse domains. Experimental results on three public benchmark datasets (OfficeHome, PACS, and VLCS) demonstrate that our proposed algorithm achieves an improvement of up to 3 . 38*%* over existing methods in terms of generalization performance, particularly in complex and diverse real-world scenarios, such as autonomous driving and medical image analysis. These results highlight the practical utility of our approach in environments where cross-domain generalization is crucial for system reliability and safety.

## Introduction

At present, deep learning research is developing rapidly, but its application in industry is limited. Since deep learning relies heavily on an oversimplified assumption that the training and test domain data are independent and identically distributed. However, domain shift [1,2] in cross-domain transfers causes models with excellent training performance to fail in real-world scenarios. Therefore, more and more research focuses on solving this problem, especially in critical applications such as autonomous driving [3–5], medical image analysis [6–8] and seismic signals classification [9,10].

From a statistical point of view, a model is obtained by establishing a conditional probability distribution over a sample spatial distribution. When the input space’s marginal probability distribution and the task’s conditional probability distribution change, it will cause a noticeable shift in data distribution between the target and source domain. This change is called a domain shift. Domain transfer describes a problem of uneven data distribution in the source and target domains [11,12].

To address the impact of the domain shift problem, domain generalization [13–15] has become a research hotspot. More and more work tries to design unique learning methods or make better model selections to achieve higher performance. Domain invariant feature learning methods [16–18] aim to identify the invariance of associations within training domain data. The stable learning method [19–21] examines the impact of sample weights on model stability. Among them, the ensemble learning [22–24] method stands out and has become a mainstream research method of domain generalization. Ensemble learning can bypass the domain transfer problem without resorting to more data, but it also brings some problems. For example, ensemble learning is more dependent on the number of models and the quality of data, which requires many resources during training and cannot be used when computer resources are limited [25,26]. In addition, ensemble learning is not biased towards designing methods to solve domain transfer but votes through the outputs of multiple models, which is not conducive to few-shot tasks and improves the generalization performance of a single model [27,28].

In this paper, we propose a simplified self-ensemble learning method based on shared feature extractors, which requires only one model and uses different classifiers for better generalization. First, we propose using the same feature extraction network to train multiple classifiers alternately so that different classifiers learn different data and establish differences between them. Second, we introduce focal loss [29] and loss weighting methods to force the model to focus on more complex samples and learn domain-independent features of both complex and simple samples. Finally, weighted voting is performed on the prediction results of multiple classifiers according to the training accuracy weight of the classifier so that the model outputs a prediction result with the highest probability after weighted voting. Three benchmark datasets representing domain discrepancy are used for local experiments under the same hyperparameter settings. First, we design a simplified self-ensemble learning framework consisting of only one shared encoder and multiple classifiers of the same type without the need to save additional copies of the model or train specific networks for different domains during training. Therefore, low consumption of computing resources by the simplified model is guaranteed. Second, the introduction of the well-proven focal loss enables the network to focus on mining domain-invariant representations of complex samples and improves the model’s generalization performance in a targeted manner. Finally, the dynamic loss weighted voting strategy is used to dynamically adjust the respective weights of the prediction results of multiple classifiers according to the size of the training loss in this round so that the classifiers with better performance get greater weights, and the classifiers with different learning abilities are improved—the problem of deciding the prediction result according to the same weight. Our contributions can be summarized as follows:

- For the first time, we propose a simplified self-ensemble learning framework that only uses shared feature extractors and multiple classifiers, which significantly reduces computer resource consumption and avoids the problem that ensemble learning cannot be used due to resource constraints.
- We design a novel voting strategy that redistributes weights based on the predictive performance of each classifier before voting and uses the average result of weighted summation as the final output.
- We propose to use focal loss and cross-entropy to weigh the loss of complex samples to improve the model’s learning ability for complex samples, making it more conducive to learning domain-invariant representations.
- We demonstrate the reliability of the proposed algorithm by thoroughly investigating the domain generalization problem under natural and synthetic images on three benchmark datasets with different settings.

The Introduction section outlines the research background and objectives of the Simplified Self-Ensemble Learning (SSEL) approach. The Related Works section reviews theoretical work on domain generalization and ensemble learning. The Methods section presents the research problem, network model design, loss function construction, and training strategy. The Experiments section details the dataset, experimental baselines, and implementation. The Results section focuses on experimental results, while Analysis Section analyzes these results and related findings. Finally, Conclusion section concludes with key findings and future research directions.

## Related works

### Domain generalization

The goal of domain generalization is to generalize a model learned from a source domain to an arbitrary new target domain. The training and testing domains do not belong to the same domain. Unlike the domain adaptation problem, domain generalization does not give a specific target domain. Domain generalization aims to have a robust generalization performance when facing any unknown target domain. The current popular domain generalization methods include domain-invariant representation learning, data augmentation, meta-learning, and regularization. Methods based on domain-invariant representation learning aim to use models to learn domain-invariant features that are not perturbed by domain-specific representations, and typical approaches use adversarial learning to try to separate domain-invariant and domain-specific representations. For example, Nguyen *et al.* [30] proposed to learn domain-invariant representations using domain density transformation theory to force the representation network to remain invariant under transformation functions in arbitrary domains. Hu *et al.* [31]proposed to achieve domain-specific feature adversarial decoupling through maximum-minimum disentanglement to learn domain-invariant representations in the task of person re-identification. Hou *et al.* [32] designed a BatchFormer module to learn sample associations in each mini batch.Lee *et al.* [33] proposed Multi-EPL, a multi-source domain adaptation method that uses label-wise moment matching and an ensemble of feature extractors to learn robust, domain-invariant representations. The method based on data augmentation enables the model to learn a broader range of features by enhancing the feature diversity of the existing data and alleviates the problem of poor model generalization ability due to the limitation of data diversity. Mancini *et al.* [34] proposed to mix images and features for data augmentation during training simultaneously. Li *et al.* [35] proposed a randomized feature enhancement method based on Gaussian noise perturbation features to train the generalization model. Xu *et al.* [36] proposed data augmentation by linearly interpolating between two images’ Fourier transform magnitude spectra.Jiang *et al.* [37] proposed MeshCut, a data augmentation technique utilizing mesh masks to diversify image features and enhance model generalization. Recently meta-learning-based methods were introduced into DG. The meta-learning involved in DG mainly simulates the distribution difference between the training domain and the test domain by constructing the meta-training domain and the meta-test domain during the model training process so that the model actively learns how to deal with the domain transfer in the unknown domain. For example, Finn *et al.* [38] proposed to use the first derivative method to compute meta-learning. Li *et al.* [39] trained the network by simulating meta-training data and meta-testing data. Shu *et al.* [40] proposed a new Dir-mixup method and distilled soft-labeling to enhance each domain and perform meta-learning accordingly. Zhao *et al.* [41] proposed a memory-based multi-source meta-learning strategy to solve the person re-identification problem.Xue *et al.* [42] leveraged meta-learning for efficient GFRP column data modeling with limited samples. Methods based on regularization strategies are often used in conjunction with other methods, such as meta-learning*et al.* [43,44], data augmentation*et al.* [45,46], and domain-invariant representation learning*et al.* [47,48].

### Ensemble learning

Ensemble learning has always been a hot topic in machine learning. Ensemble learning methods usually employ a combination of multiple copies of the same model with different weights or training data for ensemble prediction. This straightforward technique is very effective in improving model generalization [49,50]. The mainstream methods for applying ensemble learning in DG problems include ensemble SVM classifiers [51–53], domain-specific networks [54–56], and weight averages [57]. However, integrating SVM classifiers requires integrating different types of classifiers. Domain-specific networks require learning a separately used specific network for each domain. Weight averaging requires training multiple copies of the model and then averaging the weights of different models before merging. These existing methods have problems such as excessive resource consumption, repeated training, and not paying attention to complex local samples. Our proposed method does not need to train multiple copies repeatedly, only uses one encoder and multiple classifiers of the same type to achieve ensemble learning, and focuses on the case of locally complex samples.

## Methods

### Problem definition

Given *X* be the input feature space and *Y* be the label space. The domain is defined as the joint probability distribution $P_{XY}$ over *X* × *Y*. For a given $P_{XY}$, we denote the marginal probability distribution over *X* by $P_{X}$. Given *X*, $P_{Y|X}$ represents the posterior probability distribution of *Y*. $P_{Y|X}$ represents the posterior probability distribution of *Y* given *X*. $P_{X|Y}$ represents the conditional probability distribution of *X* given *Y*. The deep learning model is defined as *F* : *X* → *Y*. In domain generalization, we assume that *N* different source domains are accessible. Given the source domain as $D_{s}={\left\{D_{i}\right\}}_{i=1}^{N}$ and the target domain by $D_{t}={\left\{D_{j}\right\}}_{j=N+1}$. Each source domain obeys a joint probability distribution by $P_{XY}^{\left(n\right)}$. In general, $n\neq n^{\prime }$, $n,n^{\prime }∋\{1,\ldots ,N\}$, $P_{XY}^{\left(n\right)}\neq P_{XY}^{\left(n^{\prime }\right)}$. The labels of the source domain are accessible during model training, but the labels of the target domain are not. The goal of DG methods is to learn a model *F* with well-generalized ability on *N* visible source domains, which can achieve minimum prediction error on unseen target domains, It can be expressed as:


min ⁡ fE (xis,yis)∈Ds[L(f(xis),yis)], (1)

where *E* denotes the expectations, *L* ( ⋅ )  represents the loss function.

### Model structure

**Fig 1:**
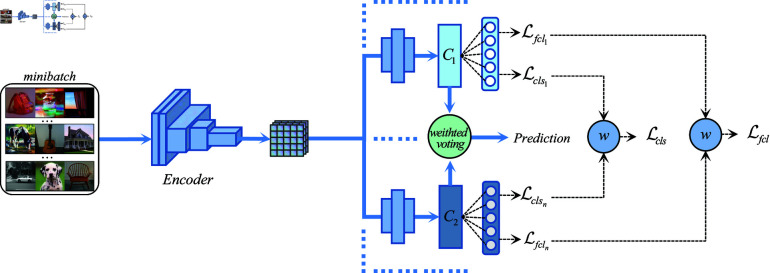
The overview of our proposed SSEL model structure. *C* ( ⋅ )  denotes different independent classifier heads. *W* represent the process of dynamic loss weighting.

Fig 1 shows our proposed model framework. The network consists of an encoder (*Encoder*) and *n* classifiers ($C_{i}$) and convolution modules belonging to different classifiers. Among them, the encoder is shared by the classifiers, and different classifiers learn different parameters by setting the random training convolution module during training. In theory, the encoder can be substituted with various feature extraction networks, including ResNet, DenseNet, EfficientNet, MobileNetV2, RegNet, and Vision Transformer, among others. This paper consistently adheres to the experimental settings established by DomainBed and employs ResNet-18 and ResNet-50 as encoders for the subsequent experiments. The number of classifiers can be set arbitrarily and within a limited range. The effect of ensemble learning will increase with the increase in the number of classifiers. Similar to the principle of diminishing marginal returns, having an excessive number of classifiers can adversely affect the model’s performance. It is worth noting that, as in other benchmark experiments, the classifier used in this paper is a single-layer fully connected network.

The core of this network structure has two parts: (1) Our proposed simplified self-ensemble learning framework only require one encoder and multiple classifiers with convolutional modules to achieve self-ensemble learning and does not require additional time to train multiple replica models or save different iteration cycles. Model parameters are used for parameter averaging. (2) We design a new dynamic loss adaptive weighted voting strategy, which can reasonably assign weights according to the classifier’s performance through the iterative loss of the classifier without introducing additional artificial hyperparameters. Algorithm 1 provides the detailed training process of our methods.

To implement the proposed method, the training process begins by initializing the encoder and classifiers with random weights. During each training epoch, a mini-batch of data is passed through the shared encoder to extract feature embeddings, which are then input to each classifier to generate predictions. The loss for each classifier is computed as a weighted combination of cross-entropy loss and focal loss, with dynamic weights assigned to balance their contributions. These weights are updated iteratively based on the relative performance of the classifiers, ensuring that classifiers with lower losses (indicating better performance) are assigned higher weights. The total loss for training is obtained by summing the weighted losses across all classifiers. During inference, the dynamic loss adaptive weighted voting strategy combines the predictions from all classifiers, giving more influence to better-performing classifiers. This framework ensures computational efficiency through a shared encoder and simple classifier architecture, while effectively addressing challenging samples to improve domain generalization performance.

### Loss function

The loss function of our proposed simplified self-ensemble learning framework consists of only two parts, namely, the cross-entropy loss $L_{cls}$ and the focal loss [58] $L_{fl}$. The cross-entropy loss is used for the classifier to update the gradient correctly, and the focal loss is used to force the model to mine more complex classified samples. The core of how they work is that we propose to use only the loss to compute the global weights, letting the model dynamically adjust the appropriate weights each epoch. A dynamic adaptive loss-weighted voting strategy is used during prediction to more reasonably assign weights to each classifier more reasonably according to the change in the loss.

**Focal loss.** The focal loss $L_{fl}$ was initially proposed to address the class imbalance problem. The class imbalance makes it more difficult to classify a small number of samples than the majority. Class imbalance and difficult classification of complex samples are similar problems. We found that the class imbalance problem and the complex sample problem are common in domain generalization. Therefore, we introduce focal loss to balance the classification preferences of the classifier for different samples so that the model pays more attention to the difficult samples to classify. It can be calculated as follows:


FL ⁡  (pt)=−αt (1−pt)γ log ⁡  (pt), (2)

Where $\alpha_{t}$ denotes a weighting factor, $\alpha_{t}∋[0,1]$. $p_{t}$ reflects the degree of closeness of the prediction to the ground truth, *γ* stands for a regularization factor. **Dynamic adaptive loss weighting.** We can evaluate the performance of the classifier based on the relative size of losses, such as cross-entropy loss or focal loss. The lower the loss value, the better the classification performance, which indicates better generalization performance in the domain generalization problem. Therefore, the following calculation can be obtained:


$$\begin{array}{ccc} \omega_{L(\cdot )}^{i}=\frac{L^{i}(\cdot )}{\overset{N}{\underset{i=1}{\sum }}L^{i}(\cdot )}, & & \end{array}$$ (3)

where $L^{i}(\cdot )$ stands for the same loss of the specific classifier, $L_{cls}$ or $L_{fl}$. *i* denotes the number of classifiers.

**Total Loss.** The total loss consists of an adaptive weighted cross entropy loss and an adaptive weighted focal loss, expressed as follows:


$$\begin{array}{ccc} L_{total}=\overset{N}{\underset{i=1}{\sum }}(\omega_{cls}^{i}L_{cls}^{i}+\omega_{fl}^{i}L_{fl}^{i}), & & \end{array}$$ (4)

where $\omega_{cls}^{i}$ and $\omega_{fl}^{i}$ are dynamic adaptive loss weights determined by , respectively.

### Dynamic adaptive loss weighted voting strategy

When the loss uses adaptive weighting to assign different weights, we propose to weight the votes using the global properties of that weight. It is worth noting that, contrary to the idea of weighted loss, we need to give more weight to classifiers with smaller loss values [59]. Therefore, the dynamic adaptive loss-weighted voting strategy can be expressed as follows:


$$\begin{array}{ccc} P_{total}=\frac{1}{N}\overset{N}{\underset{i=1}{\sum }}\frac{Y^{i}(\cdot )}{\omega_{cls}^{i}}, & & \end{array}$$ (5)

where $Y^{i}(\cdot )$ represents the output of the classifier, *N* stands for the number of classifiers, $P_{total}$ denotes the final output of our simplified self-ensemble learning framework.

## Experiments

This section mainly describes the details of the experiments performed on the three datasets. In the Dataset section, we first introduce the fundamental characteristics of the three benchmark datasets: OfficeHome, PACS, and VLCS. Second, we address critical details of the experimental design in the Implementation details section. Finally, we describe and analyze the experimental results in the Analysis section.

### Dataset

The proposed method is validated on two real-world benchmark datasets and one virtual benchmark dataset, including OfficeHome, VLCS, and PACS. See below for details.

**OfficeHome** [60] is a real-world benchmark dataset that includes four domains, each of which includes 65 categories related to office supplies, with a total of 15,888 samples.

**VLCS** [61] is a common natural image benchmark dataset in domain generalization research. It includes four domains, each containing five categories of chairs, cars, birds, dogs, and people, with a total of 10,729 samples.

**PACS** [62] is another typical unnatural image benchmark dataset consisting of four domains: Sketch, Photo, Art Painting and Cartoon. Each domain includes guitar, house, horse, dog, elephant, giraffe, and person with a total of 9,991 samples.

### Baselines

We selected five well-known domain generalization algorithms for a fair comparison with the proposed algorithm to examine the performance of the proposed algorithm. (1) DANN [63] proposes using gradient inversion layers to enhance in-depth features to improve the model’s generalization ability. (2) GroupDRO [64] proposes to use Distributed Robust Optimization (DRO) combined with regularization coupling to improve generalization performance. (3) ANDMask [65] proposes uniform learning consistency and improves generalization by fusing multiple regularization strategies. (4) SagNet [66] performs domain-invariant representation learning through adversarial learning to reduce the image style bias of model learning. (5) VREx [67] proposes variance risk extrapolation to ensure generalization outside the distribution.

### Implementation details

We follow the DomainBed protocol and use the same experimental setup to ensure fair comparisons. Specifically, 80*%* of the source domain serves as the training set, the remainder as the validation set, and the complete target domain as the test set. Model selection is based on validation accuracy in the source domain, with the highest corresponding test accuracy taken as the final result. The dataset is split using ten random seeds, and the best result among these is reported. Hyperparameters are set as follows: 30 epochs, learning rate 1e-2, batch size 32, and weight decay 5e-4 for all datasets.

## Results

All experimental results described in this section are obtained from local personal PC training. We present the experimental results on the three datasets separately and give a complete analysis.

**Table 1:** Accuracy (%) on OfficeHome for domain generalization (ResNet-18).

Method	A	C	P	R	Avg
ANDMask	48.91	43.71	65.53	66.74	56.22
GroupDRO	53.07	44.77	66.01	68.51	58.09
DANN	53.69	46.07	68.35	68.10	59.05
SagNet	55.58	46.03	70.17	73.12	61.23
VREx	55.79	46.03	70.22	**73.35**	61.35
**SSEL(ours)**	**56.86**	**46.96**	**71.21**	73.01	**62.01**

**Results on OfficeHome.** Our experimental results on the OfficeHome dataset are summarized in Table 1. OfficeHome contains 65 subdomain categories, making generalization more challenging due to its diversity. Here, A denotes the target domain Art, C denotes Clipart, P denotes Product, and R denotes Real World. The training domain excludes the target domain. For A, there are 13,161 training samples and 2,427 testing samples; for C, 11,223 training samples and 4,365 testing samples; for P, 11,149 training samples and 4,439 testing samples; and for R, 11,231 training samples and 4,357 testing samples. Our method, leveraging a simplified self-ensemble learning approach and weighted voting strategy, achieves superior performance across all four domains. In Clipart, the most challenging domain, our algorithm’s accuracy surpasses the second-best (DANN) by 0 . 89*%*. On average, it outperforms the second-best (VREx) by 0 . 66*%*, demonstrating clear advantages over recent state-of-the-art methods.

**Table 2:** Accuracy (%) on PACS for domain generalization(ResNet-18).

Method	P	A	C	S	Avg
ANDMask	87.84	69.68	70.69	75.80	76.00
GroupDRO	86.77	70.75	70.82	78.09	76.61
DANN	88.26	70.65	66.00	**80.94**	76.46
SagNet	92.81	75.39	76.96	76.28	80.36
VREx	92.75	73.39	71.16	74.98	78.07
**SSEL(ours)**	**93.47**	**77.54**	**77.01**	77.78	**81.45**

**Results on PACS.** Our experimental results on the PACS dataset are shown in Table 2. The PACS dataset contains many virtual images and fewer natural images, making it suitable for verifying generalization performance on synthetic images. Here P represents the target domain of Photo, A represents Art Painting, C represents Cartoon, and S represents Sketch. The training domain consists of all other domains excluding the target domain. When P is the target domain, there are 8,321 training samples and 1,670 testing samples. When A is the target domain, there are 7,943 training samples and 2,048 testing samples. When C is the target domain, there are 7,647 training samples and 2,344 testing samples. In contrast, when S is the target domain, there are 6,062 training samples and 3,929 test samples. Our method shows a generalization ability far ahead of other methods in the Photo and Art-Painting domains with the most significant domain discrepancy. When Photo is the target domain, the prediction accuracy of our method was 2 . 15*%* higher than the second place (SagNet). When Art-Painting was the target domain, the prediction accuracy of our method is 0 . 05*%* higher than the second place (SagNet). These results indicate that our method has a stronger generalization ability.

**Table 3:** Accuracy (%) on VLCS for domain generalization(ResNet-18).

Method	V	L	C	S	Avg
ANDMask	64.19	63.70	92.79	62.22	70.73
GroupDRO	65.14	59.41	92.08	66.06	70.67
DANN	63.09	62.88	92.37	57.71	69.01
SagNet	67.46	62.84	91.59	71.09	73.25
VREx	67.09	63.82	93.07	68.57	73.14
**SSEL(ours)**	**68.83**	**64.38**	**94.42**	**73.61**	**75.31**

**Table 4:** Accuracy (%) on three datasets for domain generalization (ResNet-18).

Method	OfficeHome	PACS	VLCS	Avg
ANDMask	56.22	76.00	70.73	50.74
GroupDRO	58.09	76.61	70.67	51.34
DANN	59.05	76.46	69.01	51.13
SagNet	61.23	80.36	73.25	53.71
VREx	61.35	78.07	73.14	53.14
**SSEL(ours)**	**62.01**	**81.45**	**75.31**	**54.69**

**Results on VLCS.** The VLCS dataset consists of natural images, resulting in a smaller domain gap compared to other datasets. Where V denotes the target domain VOC2007, L denotes LabelMe, C denotes Caltech101, and S denotes SUN09. The training domain consists of all other domains in the dataset, excluding the target domain. When V is the target domain, there are 7,353 training samples and 3,376 testing samples. When L is the target domain, there are 8,073 training samples and 2,656 testing samples. When C is used as the target domain, there are 9,314 training samples and 1,415 testing samples. In contrast, when S is used as the target domain, there are 7,447 training samples and 3,282 testing samples. As seen from Table 3, our method outperforms other methods and achieves the best performance in all four domains(VOC2007, LabelMe, Caltech101, and SUN09). Therefore, our method also has good performance with a slight domain gap. As demonstrated in Table 4, the simplified self-learning model that integrates a dynamic adaptive loss-weighted voting strategy continues to exhibit superior performance in cross-dataset scenarios.

## Analysis

In this section, we further analyze and discuss the significant effect of the simplified self-ensemble learning framework and weighted voting strategy to enhance the model’s generalization ability without increasing the number of models. Notably, we reveal the impact of the proposed algorithm by studying the compositional changes in the loss function and learning strategy of the proposed algorithm on different datasets. Table 5, Table 6, and Table 7 present the results of ablation experiments conducted with different numbers of classifiers across three benchmark datasets. Additionally, Table 8, Table 9, and Table 10 showcase the results of the combinatorial ablation experiments performed on the same datasets. Table 11, Table 12, and Table 13 present the results of the ablation experiments comparing the adaptive dynamically-weighted loss-voting strategy and the fixed-proportional-voting strategy across three benchmark datasets. Where $L_{cls}$ denotes the cross-entropy loss, $L_{fl}$ denotes the Focal loss, and $S_{wv}$ denotes adaptive dynamically weighted loss-voting strategy.

### Main findings

From the above table, we can draw the following conclusions:

**Table 5:** Ablation accuracy (%) of our method on OfficeHome using *N* classifiers for domain generalization(ResNet-18).

*N*	A	C	P	R	Avg
2	**56.86**	**46.96**	**71.21**	**73.01**	**62.01**
3	52.58	41.81	64.65	66.54	56.40
4	53.44	43.14	60.60	64.47	55.41
5	49.53	40.21	60.42	62.50	53.17

**Table 6:** Ablation accuracy (%) of our method on PACS using *N* classifiers for domain generalization(ResNet-18).

*N*	P	A	C	S	Avg
2	93.47	77.54	**77.01**	**77.78**	**81.45**
3	94.01	74.71	75.26	77.73	80.43
4	**95.93**	**78.03**	67.86	77.07	79.72
5	92.75	76.03	66.89	76.88	78.14

**Table 7:** Ablation accuracy (%) of our method on VLCS using *N* classifiers for domain generalization(ResNet-18).

*N*	V	L	C	S	Avg
2	68.83	**64.38**	94.42	**73.61**	**75.31**
3	**69.85**	61.14	**96.68**	67.46	73.78
4	65.70	59.86	95.55	65.42	71.63
5	69.02	62.95	96.18	63.35	72.88

**Table 8:** Ablation accuracy (%) of our method on OfficeHome for domain generalization(ResNet-18).

$L_{\text{cls}}$	$L_{\text{fl}}$	$S_{\text{wv}}$	A	C	P	R	Avg
*✓*	-	-	53.36	45.71	68.24	70.67	59.50
*✓*	*✓*	-	54.26	46.56	71.10	72.63	61.14
*✓*	-	*✓*	52.82	44.47	69.05	71.31	59.41
*✓*	*✓*	*✓*	**56.86**	**46.96**	**71.21**	**73.01**	**62.01**

(1) Experiments on three benchmark datasets reveal that the model performs best when *N* = 2 classifiers are used. Using fewer high-quality classifiers improves generalization and accuracy by reducing noise and complexity. For example, experimental results on OfficeHome show that average accuracy is 62 . 01*%* at *N* = 2, dropping to 56 . 40*%* at *N* = 3, 55 . 41*%* at *N* = 4, and 53 . 17*%* at *N* = 5. While increasing the number of classifiers may reduce overfitting on some datasets, adding low-quality or redundant classifiers often introduces noise, degrading performance. On PACS, average accuracy peaks at 81 . 45*%* (*N* = 2) but decreases to 78 . 14*%* (*N* = 5). Similarly, in weighted voting, low-quality voters overshadowing high-quality ones can reduce decision accuracy. On VLCS, this leads to a decline in average accuracy from 75 . 31*%* (*N* = 2) to 72 . 88*%* (*N* = 5). These results highlight that two high-quality classifiers are sufficient to achieve stable and accurate results while avoiding unnecessary complexity and noise. Increasing the number of classifiers does not necessarily enhance performance; instead, it may introduce noise and lead to biased judgments in weighted voting. This is particularly evident when low-quality voters overshadow the correct assessments of high-quality ones, ultimately reducing decision-making accuracy.

**Table 9:** Ablation accuracy (%) of our method on PACS for domain generalization(ResNet-18).

$L_{\text{c l s}}$	$L_{\text{fl}}$	$S_{\text{wv}}$	P	A	C	S	Avg
*✓*	-	-	92.22	76.46	75.04	76.51	80.06
*✓*	*✓*	-	93.05	77.27	75.84	76.89	80.76
*✓*	-	*✓*	92.14	76.56	75.45	76.50	80.16
*✓*	*✓*	*✓*	**93.47**	**77.54**	**77.01**	**77.78**	**81.45**

**Table 10:** Ablation accuracy (%) of our method on VLCS for domain generalization (ResNet-18).

$L_{\text{c l s}}$	$L_{\text{fl}}$	$S_{\text{wv}}$	V	L	C	S	Avg
*✓*	-	-	67.62	62.88	91.17	66.30	71.99
*✓*	*✓*	-	70.47	62.99	94.05	68.31	73.96
*✓*	-	*✓*	67.59	62.88	93.04	68.43	72.99
*✓*	*✓*	*✓*	**73.61**	**64.38**	**94.42**	**68.83**	**75.31**

**Table 11:** Ablation accuracy (%) of weighted voting strategy on OfficeHome for domain generalization (ResNet-18).

$L_{\text{c l s}}$	$L_{\text{fl}}$	$S_{\text{wv}}$	$S_{\text{wv-fixed}}$	P	A	C	R	Avg
*✓*	*✓*	-	1:9	55.01	46.09	70.42	72.54	61.02
*✓*	*✓*	-	2:8	55.25	45.70	70.92	72.26	61.03
*✓*	*✓*	-	3:7	54.84	46.62	70.76	72.95	61.29
*✓*	*✓*	-	4:6	54.96	46.76	71.46	72.87	61.51
*✓*	*✓*	-	5:5	54.76	45.75	**71.73**	72.66	61.23
*✓*	*✓*	-	6:4	54.68	46.03	71.48	**73.15**	61.34
*✓*	*✓*	-	7:3	54.31	46.48	70.58	72.81	61.05
*✓*	*✓*	-	8:2	55.79	46.87	70.96	72.67	61.57
*✓*	*✓*	-	9:1	54.68	46.64	71.12	72.73	61.29
*✓*	*✓*	*✓*	-	**56.86**	**46.96**	71.21	73.01	**62.01**

**Table 12:** Ablation accuracy (%) of weighted voting strategy on PACS for domain generalization (ResNet-18).

$L_{\text{c l s}}$	$L_{\text{fl}}$	$S_{\text{wv}}$	$S_{\text{wv-fixed}}$	P	A	C	S	Avg
*✓*	*✓*	-	1:9	93.03	72.27	75.30	76.30	79.23
*✓*	*✓*	-	2:8	93.03	77.20	74.02	70.55	78.70
*✓*	*✓*	-	3:7	73.68	76.37	93.29	73.30	79.16
*✓*	*✓*	-	4:6	93.07	74.22	75.04	72.79	78.78
*✓*	*✓*	-	5:5	92.91	71.44	73.93	71.95	77.56
*✓*	*✓*	-	6:4	93.13	74.51	73.72	73.63	78.75
*✓*	*✓*	-	7:3	92.37	75.05	76.07	69.36	78.21
*✓*	*✓*	-	8:2	92.49	74.66	75.30	75.13	79.40
*✓*	*✓*	-	9:1	92.43	73.58	74.23	71.29	77.88
*✓*	*✓*	*✓*	-	**93.47**	**77.54**	**77.01**	**77.78**	**81.45**

(2) With only $L_{cls}$ and $L_{fl}$ included, the average precision on OfficeHome improved by 1 . 64*%* compared to when only $L_{cls}$ was included. The average precision on PACS has improved by 0 . 70*%* compared to the PACS dataset that utilized only $L_{cls}$. The average accuracy on VLCS is 1 . 97*%* higher when only $L_{cls}$ is included. The experimental results indicate that the generalization ability of the simplified self-integrated learning framework, which consists of a single encoder and dual classifiers, surpasses that of the original encoder. Moreover, the simplified self-integrated learning framework, which does not incorporate the Weighted Voting strategy, has outperformed several representative algorithms. For instance, our proposed algorithm achieves an average accuracy of 61 . 14*%* on OfficeHome, surpassing DANN (59 . 05*%*), ANDMask (56 . 22*%*), and GroupDRO (58 . 09*%*). The experimental results presented above demonstrate that the Focal Loss introduced by the second classifier is more effective in focusing on the complex samples within the training domain. These complex samples are a crucial factor in differentiating subclasses across various target domains, posing a challenge to the model’s generalization ability. The proposed simplified self-integrated learning framework demonstrates significant robustness and generalization capabilities.

(3) With the inclusion of only $L_{cls}$ and $S_{wv}$, the average accuracy on PACS improves by 0 . 10*%* compared to using only the $L_{cls}$ loss function, while the average accuracy on VLCS improves by 1 . 00*%* when using only the $L_{cls}$ loss function. The average accuracy on OfficeHome is 0 . 09*%* lower than when only $L_{cls}$ is included. The experimental results indicate that when the same cross-entropy loss is applied to multiple classifiers and the Weighted Voting strategy is implemented, the model’s generalization performance is slightly enhanced when the number of subdomains is small. When the number of subdomains in the training domain is substantial (e.g., 65 subdomains in OfficeHome), enhancing classification accuracy and generalization becomes challenging, even with the use of multiple classifiers.

(4) Incorporating $L_{cls}$, $L_{fl}$, and $S_{wv}$ significantly improves generalization performance across all scenarios (OfficeHome, PACS, VLCS). Results show that the proposed dynamic loss-weighted voting strategy enhances generalization without modifying model structure or count.

**Table 13:** Ablation accuracy (%) of weighted voting strategy on VLCS for domain generalization (ResNet-18).

$L_{\text{c l s}}$	$L_{\text{fl}}$	$S_{\text{wv}}$	$S_{\text{wv-fixed}}$	V	L	C	S	Avg
*✓*	*✓*	-	1:9	73.10	61.22	94.35	67.43	74.03
*✓*	*✓*	-	2:8	73.50	62.84	93.43	64.69	73.03
*✓*	*✓*	-	3:7	65.67	61.63	93.97	65.51	71.70
*✓*	*✓*	-	4:6	68.90	64.02	93.05	66.21	73.05
*✓*	*✓*	-	5:5	73.07	63.97	94.25	65.42	74.18
*✓*	*✓*	-	6:4	73.31	60.77	93.27	67.31	73.67
*✓*	*✓*	-	7:3	73.46	62.88	94.35	67.40	74.52
*✓*	*✓*	-	8:2	73.44	63.63	93.71	67.89	74.67
*✓*	*✓*	-	9:1	**73.87**	62.16	93.98	66.73	74.19
*✓*	*✓*	*✓*	-	73.61	**64.38**	**94.42**	**68.83**	**75.31**

(5) By setting a fixed proportion of weighted loss voting strategy between the dual classifiers ablation experiments demonstrate the advantages of adaptive dynamic weighted loss voting strategy, i.e., a fixed proportion of weighted loss voting set by human beings tends to be less effective than the weights derived from the model’s own learning. This is because artificially set hyperparameters cannot accurately guide the model in achieving generalization across different datasets.

**Fig 2:**
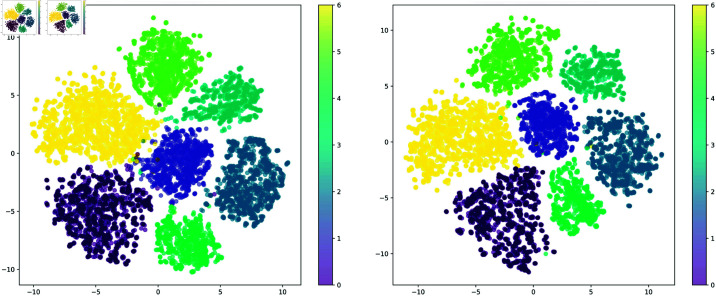
The visualization of the results employs t-SNE dimensionality reduction. The left panel presents the baseline results obtained without employing any dynamic graph (DG) algorithm, whereas the right panel demonstrates the performance of our proposed SSEL framework. Notably, the classification boundaries and inter-class distances depicted in the right panel exhibit significant enhancement relative to the baseline left panel. This comparative analysis was conducted using the PACS dataset.

In addition, the t-SNE algorithm is employed to downscale the output of the model’s final layer, resulting in the visualization presented in Fig 2. This figure demonstrates that SSEL effectively differentiates the classification boundaries between various target sub-domains, improving the confusion observed at the boundaries of the baseline algorithm. In conclusion, the results of the aforementioned ablation studies validate the model proposed in this paper. The algorithm code and dataset utilized in this study are publicly available on GitHub: https://github.com/Marzsccc.

### Limitations and future recommendations

This study has several limitations. For instance, due to hardware constraints, deeper encoder architectures that could potentially improve feature extraction were not explored, which might have limited the maximum achievable performance of the model. Furthermore, the research primarily centered on the simplified self-ensemble learning framework, leaving other complementary approaches, such as advanced data augmentation techniques and robust domain-invariant representation learning, insufficiently investigated.

In future work, we aim to address these limitations by exploring more comprehensive solutions. Specifically, we plan to investigate lightweight yet deeper encoder architectures, to integrate advanced data augmentation strategies, and to develop hybrid models that effectively combine ensemble learning with domain-specific adaptations. Additionally, we intend to evaluate the proposed framework on diverse and larger-scale datasets to ensure its broader applicability and robustness.

## Conclusion

In this paper, we propose a simplified self-ensemble learning framework and a novel adaptive weighted voting strategy with dynamic losses. The proposed framework achieves superior ensemble performance without requiring additional model copies or saving iterative parameters, indicating that a shared encoder does not compromise the generalization capability of classifiers. The dynamic loss adaptive weighted voting strategy enables different classifiers to dynamically assign the corresponding voting weights according to the loss in the training period, allowing the better-performing classifiers to gain more discourse power. As the performance of other classifiers is enhanced, the weights will still be changed relatively. In addition, by introducing focal loss, the model focuses on mining the information of complex samples and only uses the loss value to learn hyperparameters, avoiding the influence of manual parameter adjustment. Experiments on three datasets (OfficeHome, PACS, VLCS) show that our proposed algorithm achieves more robust generalization performance compared to existing methods. Experiments conducted on three benchmark datasets (OfficeHome, PACS, and VLCS) demonstrate that the proposed algorithm achieves robust generalization performance compared to state-of-the-art methods. Specifically, our method addresses two critical challenges: (1) Conventional ensemble learning methods require multiple model copies and parameter averaging, which increases computational resource demands. In contrast, our framework trains a single model, significantly reducing computational costs while maintaining generalization performance using homogeneous classifiers. (2) Existing voting strategies rarely consider dynamic weight allocation among classifiers with varying performance. Our dynamic loss-based adaptive voting strategy assigns weights adaptively based on loss variations during training. Combined with focal loss, this strategy effectively enhances the ensemble’s generalization performance. While the proposed framework demonstrates promising results, there is room for further exploration to enhance its capabilities and applicability. Future work could focus on incorporating deeper and more efficient encoder architectures to improve feature extraction and scalability to larger datasets. Additionally, advanced data augmentation strategies could be employed to diversify training data and better address domain gaps. Another valuable direction would be to integrate the framework with complementary approaches, such as meta-learning and domain-invariant representation learning, to create hybrid models that capitalize on the strengths of multiple methodologies. Furthermore, evaluating the framework in more diverse and challenging real-world scenarios, such as autonomous driving and medical image analysis, would help establish its robustness and practical value across various domains.
